# Effect of Thermal Annealing on Conformation of MEH-PPV Chains in Polymer Matrix: Coexistence of H- and J-Aggregates

**DOI:** 10.3390/polym12081771

**Published:** 2020-08-07

**Authors:** Shu Hu, Yang Liao, Yang Zhang, Xiaoliang Yan, Zhenlu Zhao, Weiqiang Chen, Xin Zhang, Hongxing Liu, Heng Li, Li Li, Ming Sun, Chuanxiang Sheng

**Affiliations:** 1 School of Electronic and Optical Engineering, Nanjing University of Science and Technology, Nanjing 210094, China; 116104000499@njust.edu.cn (S.H.); liaoyang@njust.edu.cn (Y.L.); shuyesuifengqiwu@163.com (Y.Z.); herryliang0930@njust.edu.cn (X.Y.); hengli@njust.edu.cn (H.L.); lili@njust.edu.cn (L.L.); 2 Beijing Spacecrafts, Beijing 100094, China; zhaozl529@163.com (Z.Z.); babylon75@163.com (W.C.); 13621192466@163.com (X.Z.); lhx-dd@163.com (H.L.)

**Keywords:** MEH-PPV, extended conformation, photoluminescence, J- and H-aggregates

## Abstract

In diluted solid solution using poly(2-methoxy-5-(2-ethylhexyloxy)-1,4-phenylenevinylene) (MEH-PPV) and polymethyl methacrylate (PMMA) or polystyrene (PS), both aggregated and extended conformations could be formed according to the weight ratio. Aggregated conformation in as-cast MEH-PPV/PMMA film presented a J-aggregate-like photoluminescence (PL) emission. After annealing at 160 °C, its PL showed characteristics of both J- and H-aggregates at the same time; however, extended conformation showed an oligomer-like emission, which was not sensitive to either measurement temperature or annealing temperature. Thus, the conformation transition between aggregated and extended is unlikely to happen in MEH-PPV/PMMA blends during thermal annealing. On the contrary, in MEH-PPV/PS blends, extended conformation dominated in as-cast film with oligomer-like emissions; after annealing at 160 °C, both J- and H- aggregate-like PL emissions were observed, indicating the conformation transitioned from extended to aggregated. Therefore, our work may suggest a new method to manipulate photophysical properties of conjugated polymers by combining appropriate host matrix and thermal annealing processes.

## 1. Introduction

Π-conjugated polymers (PCPs) have shown wide potentials in applications of luminescence [1,2], bio-molecular sensing [3,4], and photovoltaics [5,6]. These potentials lie not only in the near-infinite possibilities of synthesizing new materials, but also in multiple packing motifs of the polymer chains in films [7,8,9,10,11]. Furthermore, some cheap matrix polymers such as polymethyl methacrylate (PMMA) and polystyrene (PS) have proven to be useful to enhance the performance and increase the durability, while reducing the cost for light emission devices [12,13,14,15]. PCP in matrix polymers, particularly the individual PCP chain, exhibits special properties in the PL spectrum such as blinking, zero phonon lines, etc., which are absent in neat films [16,17,18,19,20]. The PL properties of PCPs in matrix polymers could be divided into two cases: aggregate-like and oligomer-like, as shown schematically in Figure S1 of Supporting Information (SI) [21]. Besides well-described intrachain J-aggregate and interchain H-aggregates [21], and because of the existence of multiple chromophores, which are a base unit for conjugation that consist of a few of monomers, we would like to emphasize another possibility of H-aggregate-like conformations within individual polymer chains [22,23]. This could result from coiled conformation of single molecules, and coiled molecules facilitate efficient exciton diffusion to low-energy sites for radiative emission through interactions between tightly stacked segments of different chromophores in single molecules [24]. For example, the coiled conformation could be taken as aggregation of multiple chromophores within a single chain [25,26,27]. Therefore, in blends of PCPs and matrix polymers, PL may be divided into two kinds: one is aggregate-like PL emissions resulting from either intrachain aggregation or aggregated chains with few interchain contacts, showing relatively lower energy (“red”) PL peaks, which is similar with that of neat film. The other is oligomer-like emissions, resulting from an extended individual chain, with higher-energy (“blue”) PL peaks, which is similar to PL from diluted solution or from oligomers consisting of few repeating units [22,24,28,29,30]. The nature of chain conformation and chain–chain interaction rely on many components such as the solvent, solution concentration, chemical composition, molecular weight of the host polymer, etc. [31,32,33], while annealing is an effective way to further manipulate its optoelectronic properties. For example, Solvent Vapor Annealing (SVA)-induced formation of aggregates for MEH-PPV/PMMA films had been demonstrated [25,34], while thermal-induced conformation transformation was also experimentally suggested [35]. However, for detailed description of chain conformation in matrix polymers after thermal annealing, in particular, the properties of aggregates which may determine the optoelectronic properties of polymer in the matrix are still lacking.

In this work, using poly(2-methoxy-5-(2-ethylhexyloxy)-1,4-phenylenevinylene) (MEH-PPV), we found there was coexistence of aggregate-like and oligomer-like emissions in MEH-PPV/PMMA (1:100 weight ratio) film (sample A), but almost only oligomer-like emissions in MEH-PPV/PMMA(1:1000) film (sample B). The as-casted sample A presented J-aggregate-like PL emissions, in which the PL 0–0/0–1 ratio increased with decreasing temperatures. After annealing at 160 °C, PL of sample A showed both characteristics of J- and H-aggregates. On the contrary, oligomer-like emissions from both samples were less sensitive to either measurement temperature or annealing temperature. However, if the host matrix changed to polystyrene (PS), no matter the weight ratio of 1:100 or 1:1000 (MEH-PPV: PS), the as-casted films contained mainly an extended conformation presenting oligomer-like emissions. After annealing at 160 °C, both J- and H- aggregate-like PL emissions were observed. Therefore, our work proved that the combination of host matrix and thermal annealing processes could adjust the photophysical properties of conjugated polymers dramatically.

## 2. Experimental Details

MEH-PPV, RR-P3HT, PS, and chlorobenzene were purchased from Sigma-Aldrich (Sigma-Aldrich Shanghai Trading Co. Ltd., Shagnhai, China). Average molecular weights of MEH-PPV and RR-P3HT were around 2 × 10^5^ and 10^5^ g/mol, respectively. PMMA, chloroform, and THF were purchased from URChem (Shanghai, China). Ten milligrams of MEH-PPV was dissolved in 10 mL different solvent (chloroform, chlorobenzene, and tetrahydro-furan) and stirred for a whole night. Ten milligrams of MEH-PPV and 10^3^ mg (or 10^4^ mg) host matrix polymer (PMMA or PS) were dissolved in 10 mL (or 100 mL) solvent mentioned above. Concentration of the RR-P3HT was 15 mg/mL obtained in the same way. Glass or CaF_2_ substrates were cleaned by ultra-sonication for 30 min in acetone and ethanol, followed by UV ozone treatment for another 30 min. The films (including the solid solution) were then drop-casted onto prepared substrates from the solution prepared above and fully dried at room temperature. These films were so-called as-cast films. In order to obtain annealed films, some as-cast films were heated at 80, 120, or 160 °C for 30 min, respectively, and then naturally cooled down to room temperature. The solution prepared was put in a quartz cuvette for optical measurement. All samples were prepared in a glove box filled with nitrogen atmosphere.

Temperature-dependent PL measurements were performed in a liquid nitrogen cooled cryostat (Janis, Woburn, MA, USA) in which the temperature could vary from 80 to 350 K. Optical excitation was carried out with a 447 nm diode laser from Changchun New Industries Optoelectronics Tech. Co., Ltd. (MDL-III-447 L) (CNI laser, Changchun, China). Absorption spectra and IR photo-induced absorption spectra were collected with a halogen tungsten lamp and monochromator (BOCI, WDG30-Z) (BOCI, Beijing, China), and a laser beam was added to be the pump for measuring IR photo-induced absorption spectra. Mid-IR photo-induced absorption spectra were measured using an IR emitter (New Port, 6363 IR) (Newport, Irvine, CA, USA), matching Zolix monochromator (Omni-λ150) (Zolix, Beijing, China) connected with Mercury Cadmium Telluride detectors (Teledyne Judson Technologies, J15 D12-M204-S01 M-60) (Teledyne Judson Technologies, Camarillo, CA, USA). PL was collected using an Idea Optics PG-4000 spectrometer (Ideaoptics, Shanghai, China).

## 3. Results and Discussion

**Figure 1:**
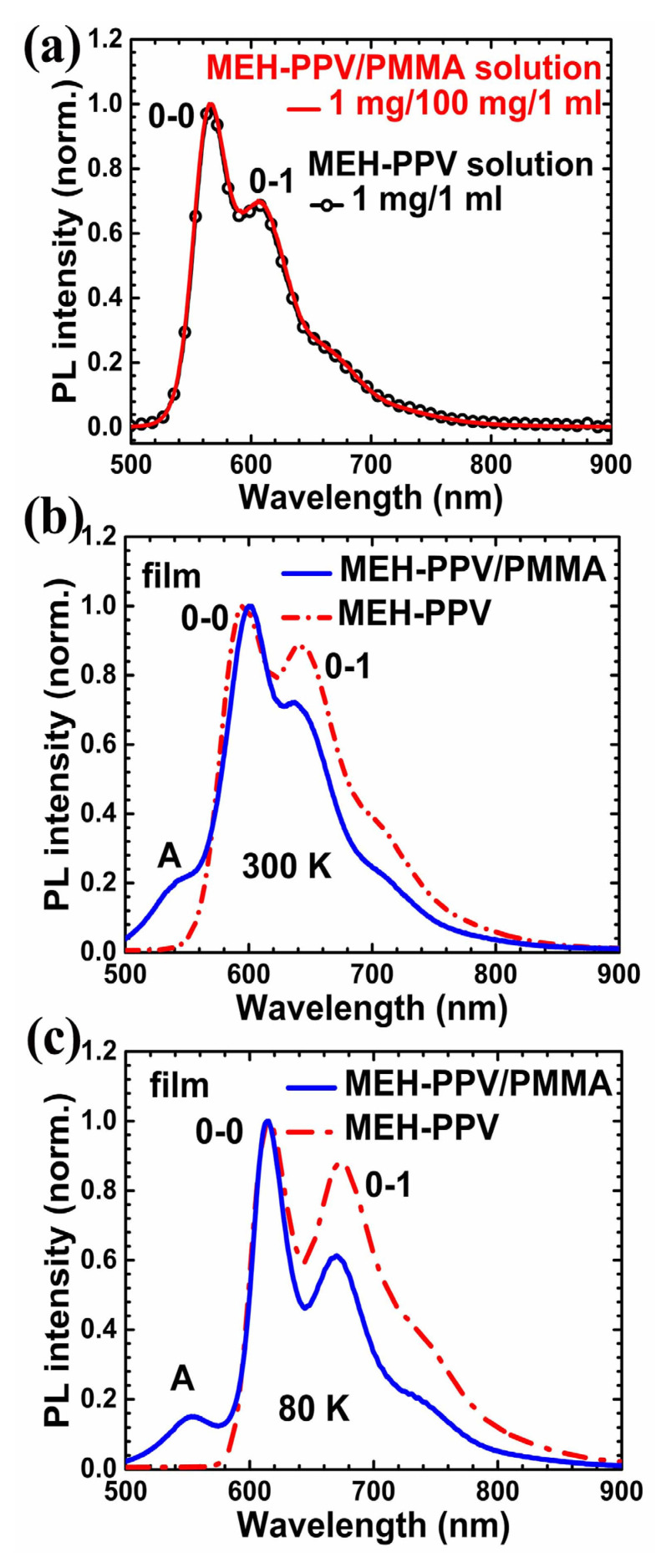
Photoluminescence spectra of MEH-PPV/PMMA and MEH-PPV. (**a**) Chlorobenzene solution measured at room temperature; (**b**,**c**) MEH-PPV/PMMA (1:100) and MEH-PPV films measured at 300 and 80 K, respectively.

In Figure 1a, we show PL spectra of two solutions, namely, MEH-PPV (1 mg) and MEH-PPV/PMMA (1 mg/100 mg) in 1 mL chlorobenzene. Identical PL spectra indicated that PMMA had no profound influence on the conformation of MEH-PPV in solution. In Figure 1b,c, PL of the films made from solutions of MEH-PPV/PMMA blends were measured at 300 and 80 K, respectively; for comparison, PL of a neat MEH-PPV film was also included. MEH-PPV chains were considered to be either isolated or slightly aggregated in solid solution of 1/100 weight ratio [28]. PL 0–0 and 0–1 transitions in blend films share similar energies with those in neat film, indicating the formation of aggregation [36], or a single polymer chain is coiled to form stacked segments of different chromophores [24]. For extended conformations of single polymer chains, such energy transfer processes between chromophores are less likely to happen; therefore, the additional emission peak at ~550 nm (this is “bluer” than the PL 0–0 transition of solution) should be due to the emission of MEH-PPV with extended conformation, described as oligomer-like emission [37,38,39,40]. We also tried different solvents including chloroform and tetrahydrofuran (THF) with the same weight ratio of MEH-PPV and PMMA. PL spectra of as-cast films from those solutions are shown in Figure S2, from which we concluded that PL of MEH-PPV in those blends from different solvents were basically neat-film-like, although the component ratio between aggregated and extended conformations should be slightly different.

**Figure 2:**
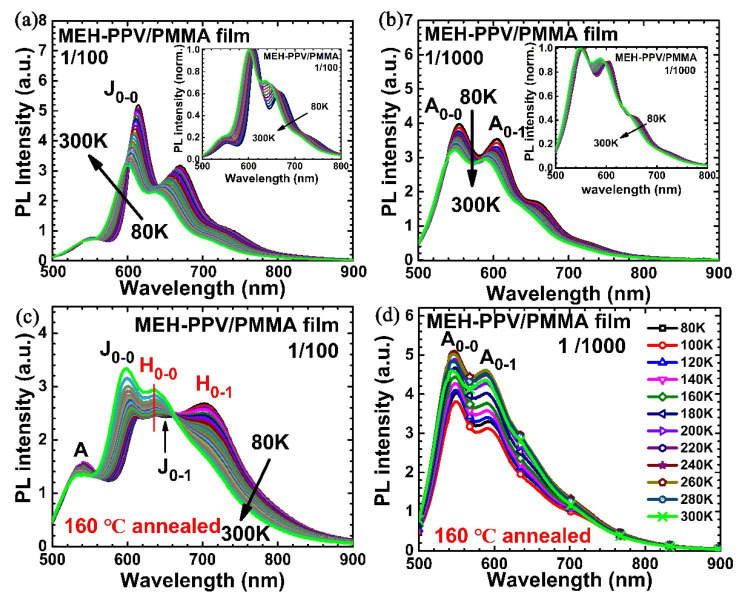
Temperature-dependent photoluminescence spectra of MEH-PPV/PMMA blends (**a**) as-cast film with weight ratio of 1/100 (sample A), (**b**) as-cast film with weight ratio of 1/1000 (sample B), (**c**) sample A annealed at 160 °C (1/100), (**d**) sample B annealed at 160 °C (1/1000). The inserts in (**a**,**b**) are the respective normalized spectra.

Figure 2a shows PL spectra at various temperatures ranging from 80 to 300 K for the as-cast MEH-PPV/PMMA (1:100) film (sample A) from its chlorobenzene solution. In Figure S3 of SI, the PL of another as-cast MEH-PPV/PMMA (1:100) film formed from a 10-fold diluted chlorobenzene solution than that for casting sample A was also included. The almost identical PL spectra and its temperature dependence indicated the same MEH-PPV conformation as in the PMMA matrix.

PL spectra of J_0–0_, J_0–1_ (J stands for J-aggregate) blue-shifts and their total intensities decreased with increasing temperatures; this is similar to neat film [41]. In Figure 2a inset, we included the normalized spectra at various temperatures. It is clear that the intensity ratio of J_0–0_ and J_0–1_ peaks increased with decreasing temperatures. In Figure S4 of SI, intensity ratios of PL J_0–0_ and J_0–1_ up to 200 K were presented after fitting the PL spectrum with four Gaussian functions. Scaling of the 0–0/0–1 PL ratio with the inverse square root of temperatures was considered as the spectral characteristic of J-aggregates [42,43]. Therefore, the J-aggregates and corresponding intrachain excitons dominated the photoexcited process in MEH-PPV/PMMA-blended film in Figure 2a and Figure S3.

For comparison, Figure 2b shows PL of blend films from 1 mg/1000 mg solution of MEH-PPV/PMMA (sample B). There are two main PL peaks around 553 nm (2.242 eV) and 604 nm (2.054 eV), labeled as A_0–0_ and its first phonon replica of A_0–1_, respectively. A_0–0_ was consistent with peak A in Figure 1b, resulting from extended conformation of isolated MEH-PPV in the PMMA matrix. It is clear that A peak was less sensitive to the measurement temperatures compared to the J-aggregate; this is consistent with its oligomer-like properties [37,38].

After annealing at 160 °C, which is above the glass transition temperature (*T*_g_~106 °C) of PMMA [44], the PL spectra of the annealed film (sample A with weight ratio of 1:100) at various temperatures from 80 to 300 K is shown in Figure 2c. For completeness, the absorption of as-cast, annealed blend films and a neat film are also shown in Figure S5 of SI. The slight blue-shift of absorption maximum in both blends films was consistent with its nature of isolated polymer chains [45]. On the other hand, annealing indeed broadens the absorption peak of blend films; however, we noted that the absorption maximum did not shift compared to the as-cast one, which confirmed that the majority of MEH-PPV chains in sample A are isolated, or at least much less aggregated, compared to neat film, otherwise the thermal annealing would lower its absorption maximum as it did in neat film (Figure S5b). Normally, the annealed neat films showed less features and red-shifted PL spectra than that of as-cast ones [46]. However, annealed blend films here presented several different features. Firstly, besides the relatively weak PL peak A, there are three obvious emission peaks in Figure 2c, including a peak near 600 nm, which is at the same energy of J_0–0_ shown in Figure 2a and has blue-shifts with increasing temperatures. Therefore, we named it as J_0–0_, ascribing to the emission of intrachain excitons of J-aggregates. This is unlike the PL spectrum of the annealed neat MEH-PPV film in which the J-aggregate-like behavior could not be detected [41]. Secondly, there were another two peaks at 637 nm (1.947 eV) and 701 nm (1.769 eV), which, however, cannot be ascribed to phonon replicas of J_0–0_. At 80 K, J_0–0_ peaked at 610 nm (2.033 eV). The energy difference (95 meV) between the peak at 637 nm and J_0–0_ was far below the normal value (~170 meV) between 0–0 and 0–1 transitions in MEH-PPV [41]. More importantly, those two peaks did not shift with the sample’s temperature as J_0–0_ did. In our previous work, we noted the annealed film presented PL 0–0 transition of H-aggregation around 630 nm, and it was also not sensitive to the measurement temperature [41]. Moreover, thermally active behavior happened, namely the intensity ratio between 637 and 701 nm peaks increased with increasing temperatures, which is a typical characteristic of PL in H-aggregated polymers. Therefore, we named two peaks at 637 and 701 nm as H_0–0_ and H_0–1_, respectively, where H stands for H-aggregate (Figure 2c). Here we would like to point out that in PL of regioregular poly (3-hexythiophene) (RR-P3 HT) film, which is the model polymer for H-aggregation, PL transition energies were also insensitive to the measurement temperatures (see Figure S6). Furthermore, 0–1 transition of J-aggregate, J_0–1_, also existed; however, it was overlapped by emissions of H-aggregation. In Figure S7, the J_0–1_ around 660 nm is clearly shown at 150 K (e.g., J_0–0_ 609 nm (2.036 eV), J_0–1_, 658 nm (1.885 eV)). Thirdly, the intensity of J_0–0_ and the ratio between J_0–0_ and H_0–0_ also increased with increasing temperature, indicating the effective energy transfer between H-aggregate and J-aggregate [47]. On the contrary, the diluted solid solution film (1:1000) did not show such dramatic changes after annealing at 160 °C (Figure 2d), indicating extended conformation was not influenced profoundly by the thermal annealing process in MEH-PPV/PMMA blends.

**Figure 3:**
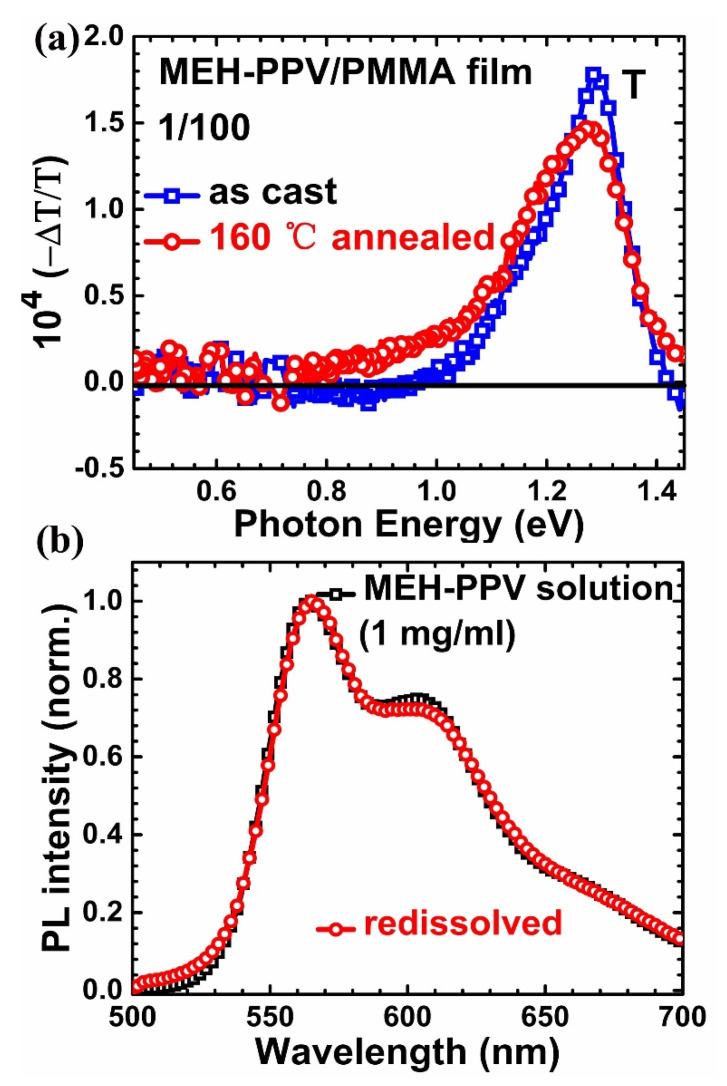
(**a**) Photo-induced absorption spectra of as-cast (red circle) and annealed at 160 °C (blue square) MEH-PPV/PMMA (1:100 weight ratio) blend films, respectively. (**b**) Photoluminescence spectra of MEH-PPV solution (black square) and re-dissolved solution of annealed blend film (red circle).

However, thermal annealing may generate defects in polymer chains. If excitons were trapped by defects, the temperature insensitivity of PL peaks or thermal activation behavior in PL spectra were also possible [48,49]. To rule out this possibility, we applied photo-induced absorption (PIA) spectroscopy in near-IR for as-cast and annealed blend films, as shown in Figure 3a, respectively. Because of the absorption of PMMA, mid-IR PIA spectra below 0.4 eV for infrared-active vibration (IRAV) modes were hard to detect. Nevertheless, we did not observe prominent polaron transition near 0.45 eV in annealed MEH-PPV/PMMA blend films [50]. Therefore, the PIA spectra prove that thermal annealing here did not generate many defects like UV illumination did [51,52]. On the other hand, the annealed film presented a broader PIA band, consistent with its absorption spectrum in Figure S5. Furthermore, in Figure S8, the identical PL spectra of blend films at various excitation intensities, and almost linear relation between luminescence intensity (*I_PL_*) and power of the excitation laser (*I*) at RT and 80 K, supplied additional evidence that the emission shown in Figure 2 is from excitons [53]. In addition, we re-dissolved the 160 °C annealed MEH-PPV/PMMA blend films using chlorobenzene, which forms much more diluted solution than original one. The PL is shown in Figure 3b, which is almost identical to the PL of the original solution, indicated no damage to the MEH-PPV chain after annealing at 160 °C.

**Figure 4:**
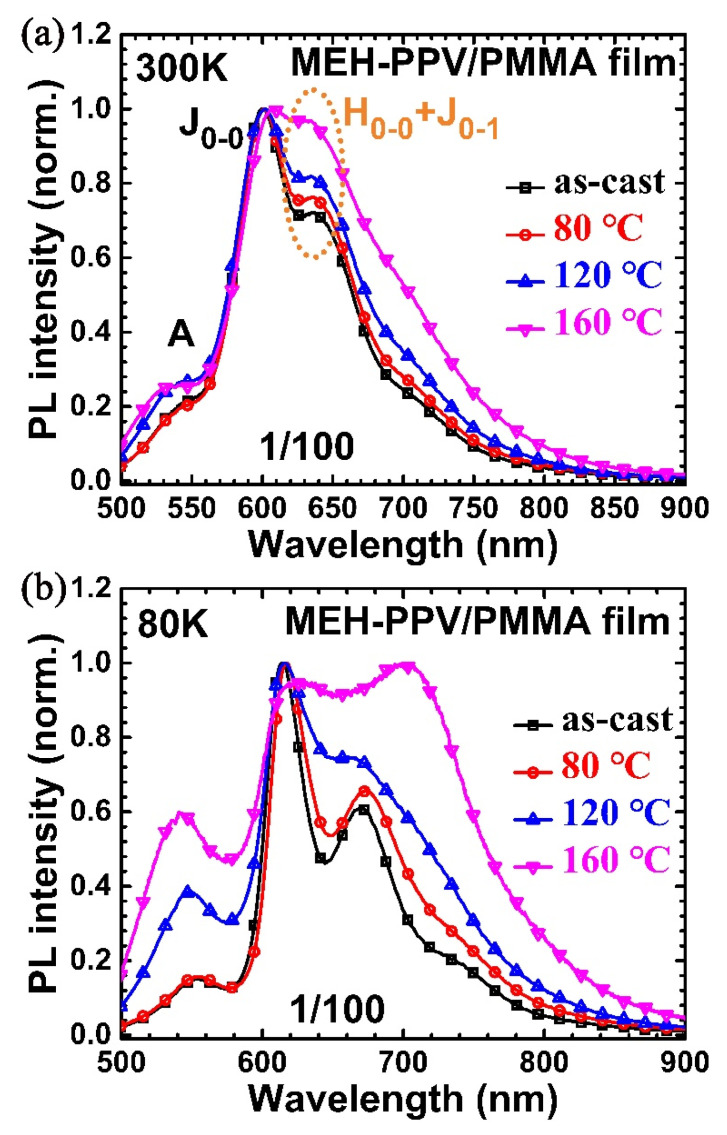
Photoluminescence spectra measured at 300 K (**a**) and 80 K (**b**) of MEH-PPV/PMMA (1/100) films of as-cast and annealed using various temperatures (80, 120, 160 °C).

For completeness, we also annealed MEH-PPV/PMMA blend films at various temperatures. Figure 4a,b shows PL spectra of those films measured at 300 and 80 K, respectively. (Complete temperature dependent PL spectra for blends annealed at 80 and 120 °C are included in Figure S9). It is clear that the relative intensity of oligomer-like emission (peak A) and the ratio between H-aggregate and J-aggregate increased with increasing annealing temperatures, suggesting that the conformations of MEH-PPV in PMMA are controllable by annealing. In addition, we tried to anneal blend films from other solvents such as from THF; its PL spectra also contained H-and J-aggregate emissions (Figure S10).

**Figure 5:**
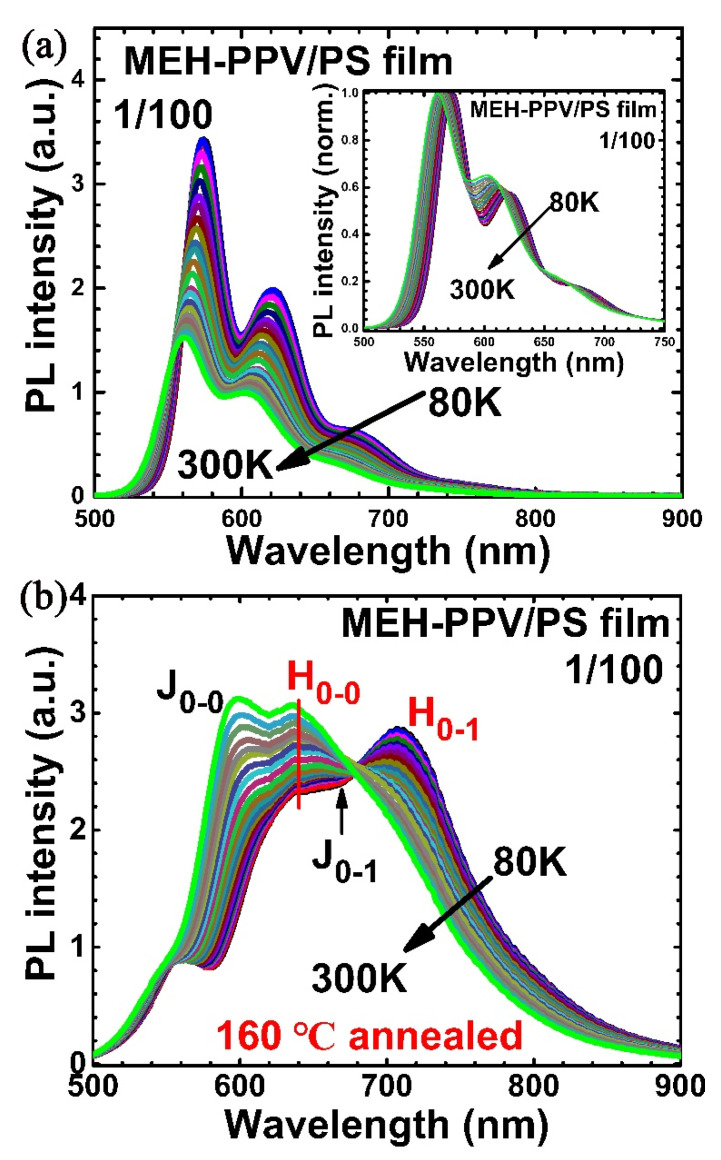
Temperature-dependent photoluminescence spectra of MEH-PPV/PS blend films with the weight ratio of 1/100. (**a**) As-cast film, (**b**) film annealed at 160 °C. Insert of (**a**) is its normalized spectra.

Another common host polymer for solid solution is PS, in which the MEH-PPV chain could be more extended [20]. In Figure 5a, we show the PL of MEH-PPV blend films from a solution of 1:100 weight ratio MEH-PPV and PS at various temperatures, where the inset shows its normalization. We noted immediately that the 0–0 transition of PL spectra shown in Figure 5a was at 580 nm, which is consistent with the expectation of an extended conformation of PCP chains [21]. After annealing at 160 °C, which is also higher than the glass transition temperature of PS (~96 °C [14]), PL spectra changed dramatically containing both J- and H-aggregate-like emissions, shown as J_0–0_, H_0–0_, H_0–1_ and J_0–1_ in Figure 5b. At the same time, thermal activation in PL spectra, i.e., effective energy transfer between H- and J-aggregate also existed in MEH-PPV/PS blends. We also used films with 1:1000 weight ratio of MEH-PPV/PS, which behaved like the 1:100 one after annealing (Figure S11). Therefore, in MEH-PPV/PS blend films, the thermal annealing would transit extended conformation to the aggregated one, which is unlikely to happen in MEH-PPV/PMMA blends.

Although conformation of PCP chains in the host polymer in PS and PMMA depends on many factors including the molecular weight of the host, solvent, and concentration [24,54], it has been extensively suggested that PMMA is a poor solvent matrix and PS is a good one; therefore, polymer chains can form more extended conformation in PS than that in PMMA [33,55] with the same concentration. This is consistent with current measurements in blends with a concentration of 1:100 of MEH-PPV in PMMA and PS, respectively. On the other hand, in a good solvent matrix, a polymer chain expending to maximize the number of solvent matrix contacts was expected, while in a poor solvent matrix, the PCP chain would minimize interaction with the solvent matrix. Although the detailed mechanism for conformation transition in blend films is out of the scope of the current work, we would like to suggest that the interaction between the PCP chain and matrix solvent is one of the crucial factors.

## 4. Conclusions

In this work, using MEH-PPV and PMMA or PS, we found that the conformation of MEH-PPV in solid solution could be aggregated (both intrachain and interchain) and extended in MEH-PPV/PMMA (1:100 of weight ratio) film, but almost only be extended one in MEH-PPV/PMMA (1:1000) film. The aggregated conformation in as-cast MEH-PPV/PMMA (mainly in 1:100) presented J-aggregate-like PL emissions, which was similar with as-cast neat film. After annealing at 160 °C, PL of MEH-PPV/PMMA (1:100) showed both characteristics of J-aggregate and H-aggregate. This was different with neat film, which lacked J-aggregate-like emissions after such annealing. On the contrary, the extended conformation in both MEH-PPV/PMMA blends presented an oligomer-like emission, which was not sensitive to either measurement temperature or annealing temperature. Thus, the transition between the aggregated conformation and the extended one was unlikely to have happened in MEH-PPV/PMMA blends during thermal annealing. However, if the host matrix was changed to polystyrene (PS), no matter the weight ratio of 1:100 or 1:1000 (MEH-PPV: PS), the as-cast film contained mainly an extended conformation and presented oligomer-like emissions. After annealing at 160 °C, both J- and H-aggregate-like, as well as relatively weak oligomer-like emissions were observed, proving the conformation transition from extended to aggregated in MEH-PPV/PS blends during thermal annealing. Therefore, selection of host matrices with an appropriate annealing processes can adjust the photophysical properties of conjugated polymer chains profoundly. Our work may supply another way to manipulate the optoelectronic properties of PCPs.

## Supplementary Materials

The Supplementary Materials are available online at https://www.mdpi.com/2073-4360/12/8/1771/s1.
