# Supplementary material for: Effect of Thermal Annealing on Conformation of MEH-PPV Chains in Polymer Matrix: Coexistence of H- and J-Aggregates
Source: Polymers (Basel). 2020 Aug 7;12(8):1771. doi: 10.3390/polym12081771 (PMC7465953; doi:10.3390/polym12081771)
Supplement: Supplementary file 1 [file polymers-12-01771-s001.pdf]

Supporting Information for the manuscript:

Effect of thermal annealing on conformation of MEH-PPV chains in polymer matrix: coexistence of H- and J- aggregates

S. Hu [a], Y. Liao [a], Y. Zhang [a], X.L. Yan [a], Z.L. Zhao [b], W.Q. Chen [b], X. Zhang [b],

H.X. Liu [b], H. Li [a], L. Li [a], M. Sun [a, *], C.X. Sheng [a, *]

[a] *School of Electronic and Optical Engineering, Nanjing University of Science and Technology, Nanjing 210094, China*

[b]*Beijing Spacecrafts, Beijing 100094, China*

Corresponding Author

**E-mail: cxsheng@njust.edu.cn (C.X.S.)；msun@njust.edu.cn*

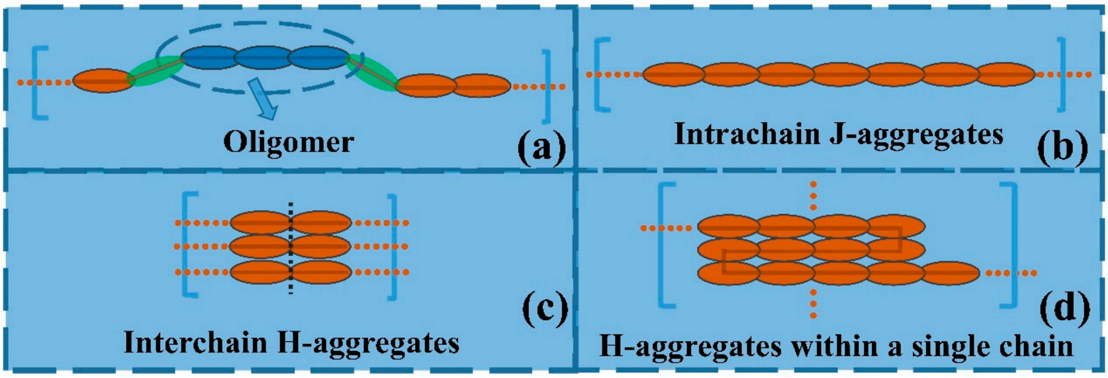

Fig. S1 Schematics of (**a**) oligomer in which the wave-function of excitons would be confined within few of repeat units. (**b**) J-aggregate within a single polymer chain. (**c**) Interchain H aggregates resulting from chain-chain interaction. Because the existence of intrachain interaction, the so-called HJ-aggregates is also possible. (**d**) H-aggregates formed within a single chain between tightly stacked segments of different chromophores.[1]

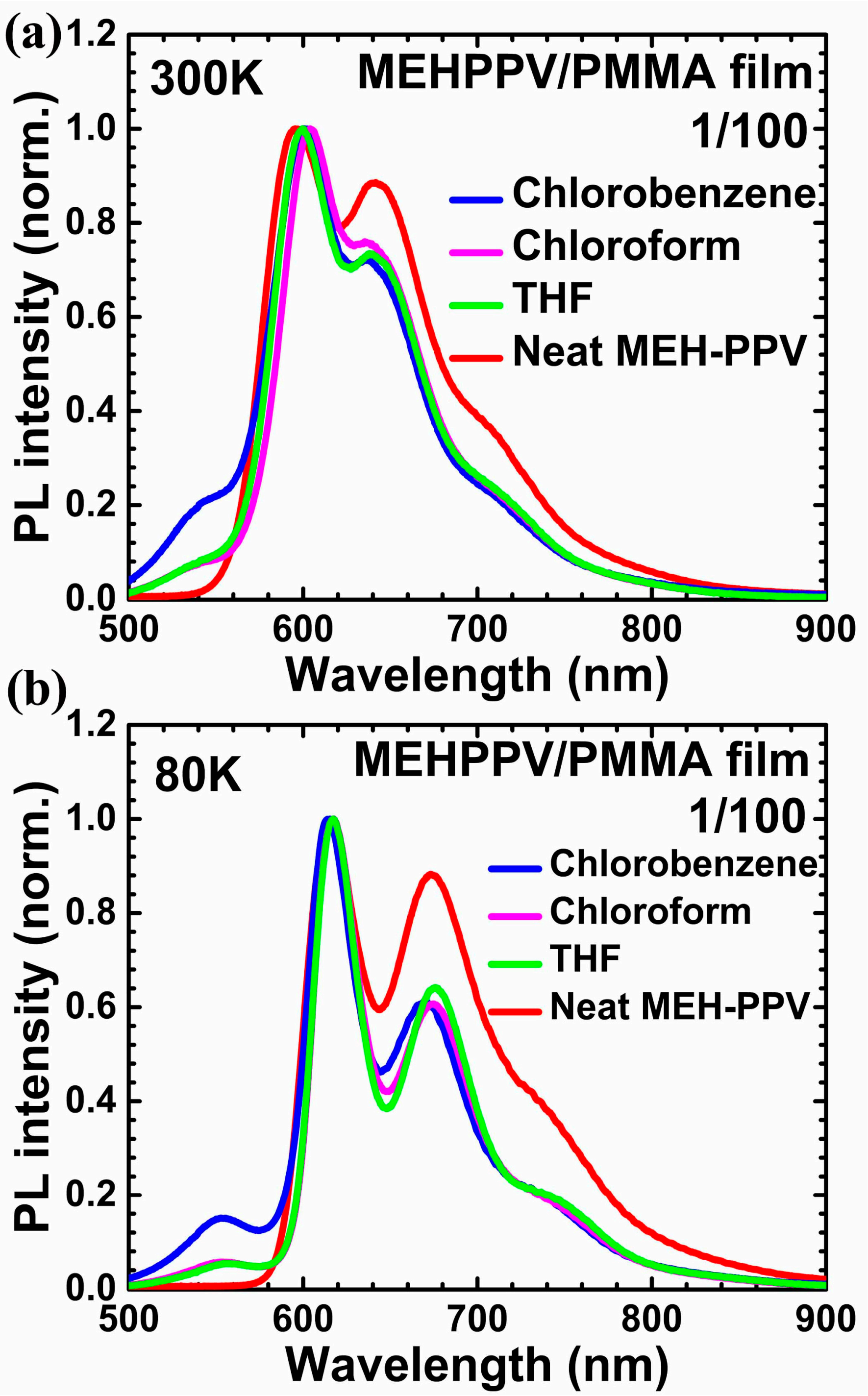

Fig. S2 Photoluminescence spectra of MEH-PPV/PMMA blends films (casted from different solvent solution) with the weight ratio is 1/100 measured at (a) 300 K (b) 80 K.

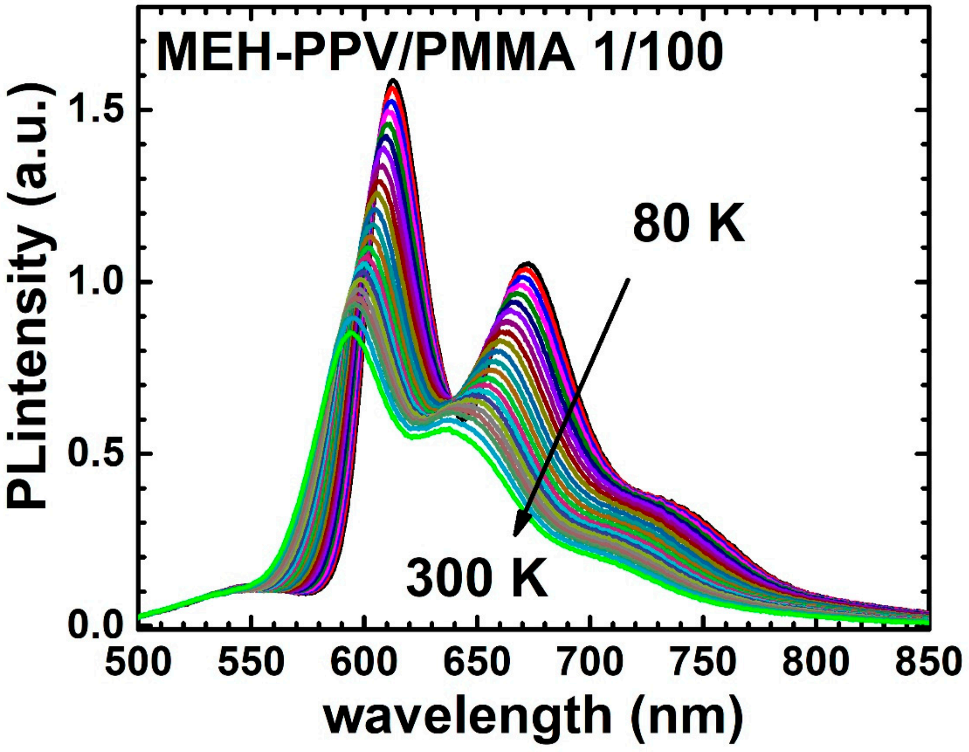

Fig. S3 Temperature dependent photoluminescence spectra of MEH-PPV/PMMA blend film with weight ratio of 1/100, from a chlorobenzene solution 10 time diluted than which was used to cast Sample A.

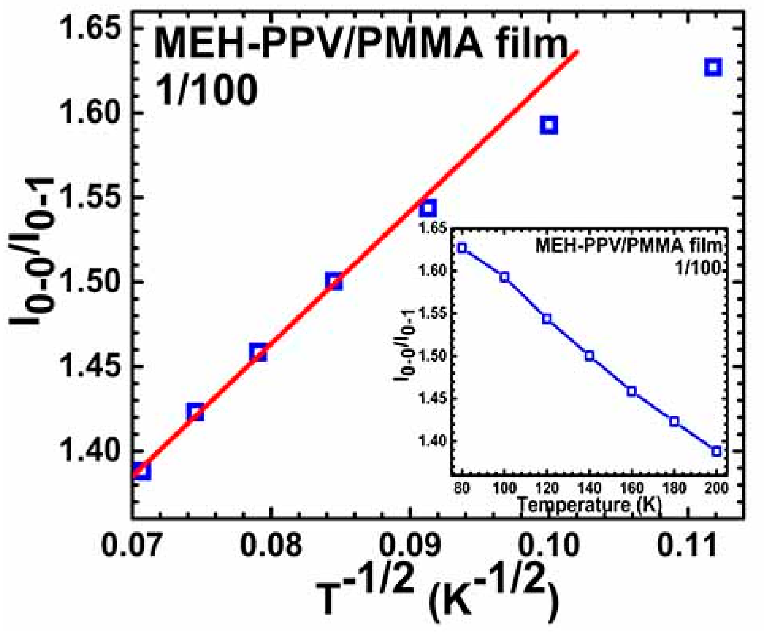

Fig. S4 The relation between inverse square root of temperature and ratio of $I_{0\text{-}0}/I_{0\text{-}1}$. Insert is the ratio of $I_{0\text{-}0}/I_{0\text{-}1}$ at different temperatures.

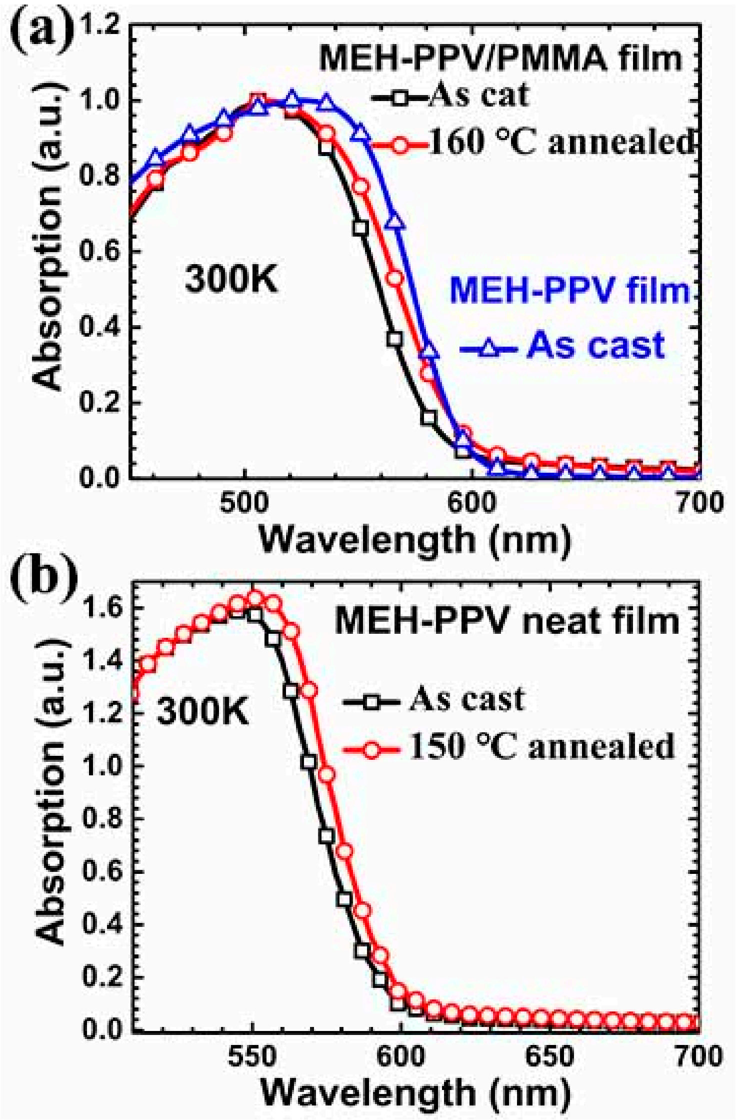

Fig. S5 Absorption spectra of (**a**) MEH-PPV film and MEH-PPV/PMMA (1/100) blend film as cast and annealed at 160 °C (**b**) MEH-PPV film as cast and annealed at ~150 °C.

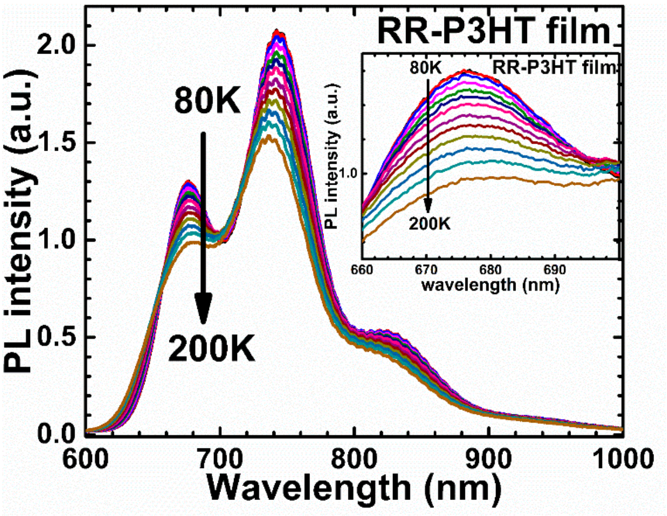

Fig. S6 Temperature dependent photoluminescence spectra of RR-P3HT film. Insert is the zoom-in of spectra with the wavelength from 660 nm to 690 nm (0-0 transition).

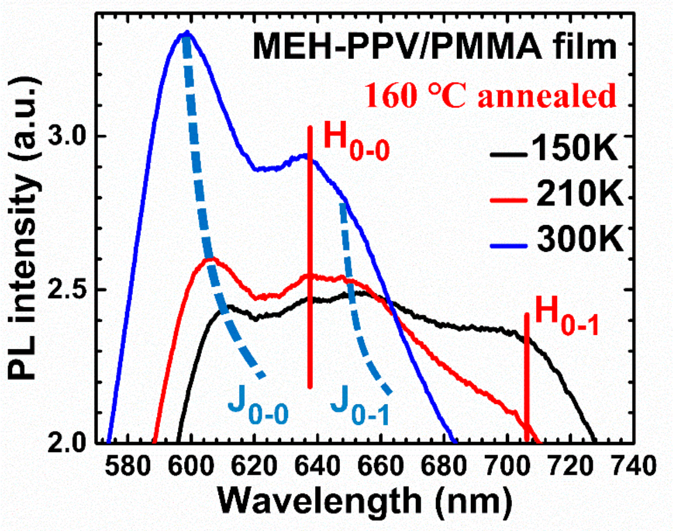

Fig. S7 Zoom-in of the photoluminescence spectra of the MEH-PPV/PMMA (1/100) blend film annealed at 160 °C measured at various temperatures. J_{0-1} transition can be discerned in clarity.

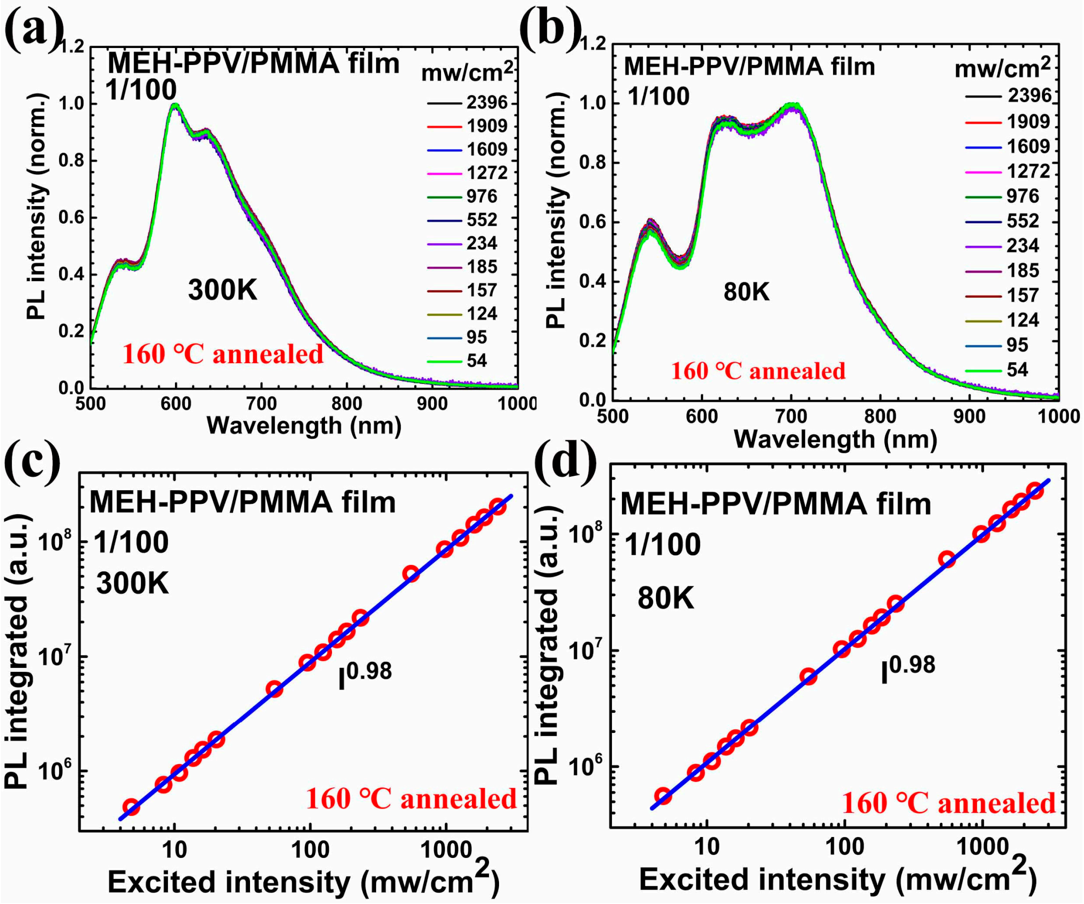

Fig. S8 The normalized photoluminescence spectra of MEH-PPV/PMMA blends film annealed at 160 °C excited with different power measured at (a) 300 K and (b) 80 K. Photoluminescence intensity as a function of the excitation laser power measured at (**a**) 300 K and (**d**) 80 K.

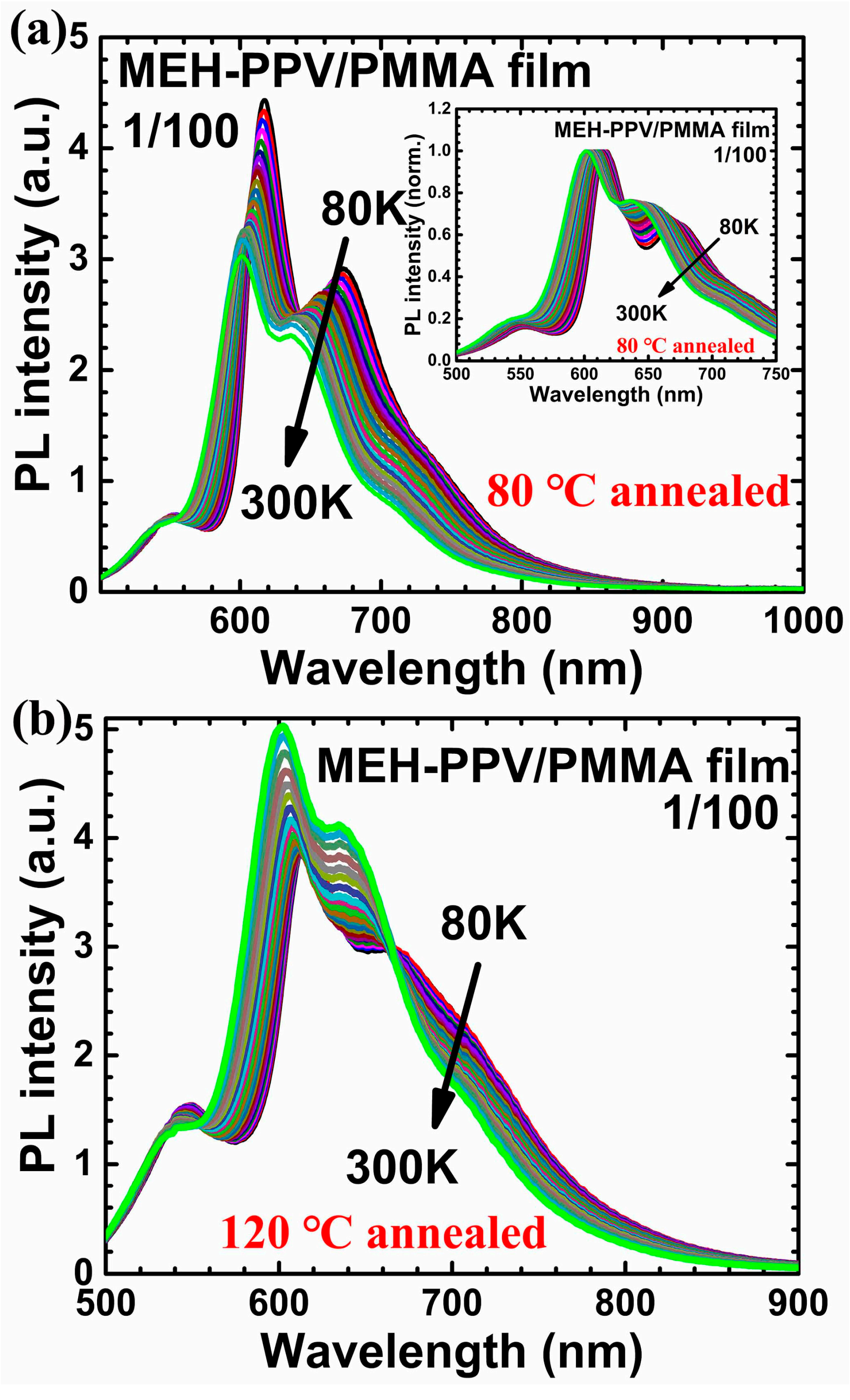

Fig. S9 The temperature dependent photoluminescence spectra of MEH-PPV/PMMA blend film (**a**) annealed at 80 °C, (**b**) annealed at 120 °C.

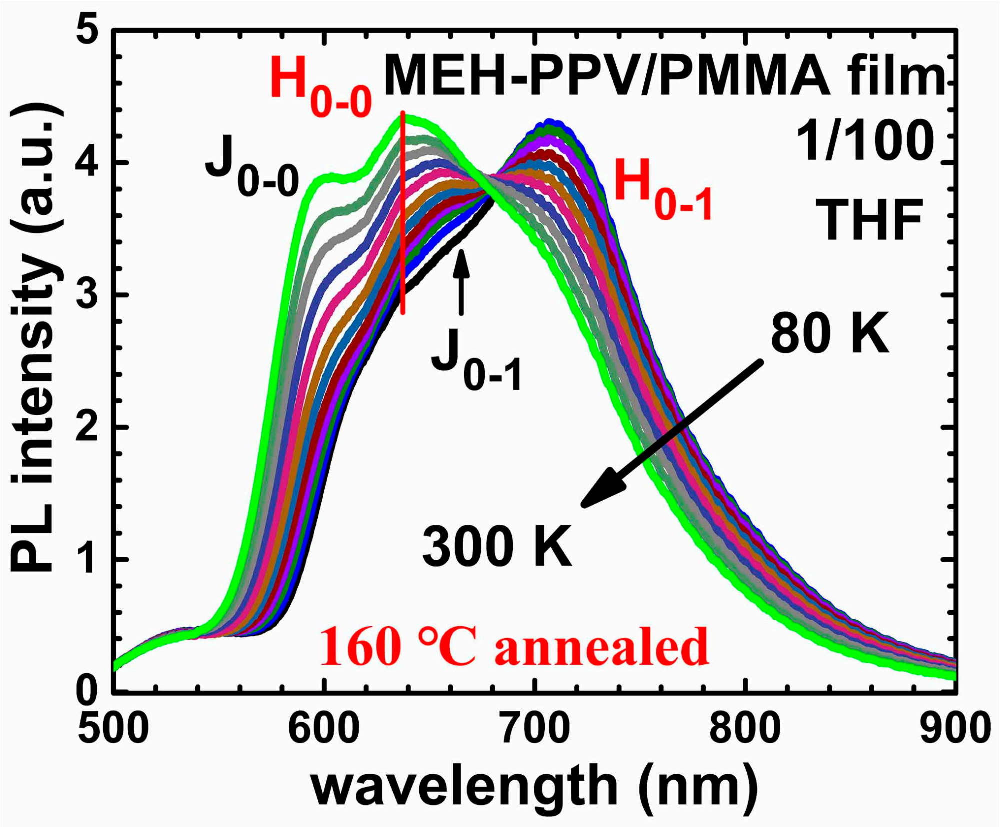

Fig. S10 The temperature dependent photoluminescence spectra of MEH-PPV/PMMA blend films generating from THF dispersions.

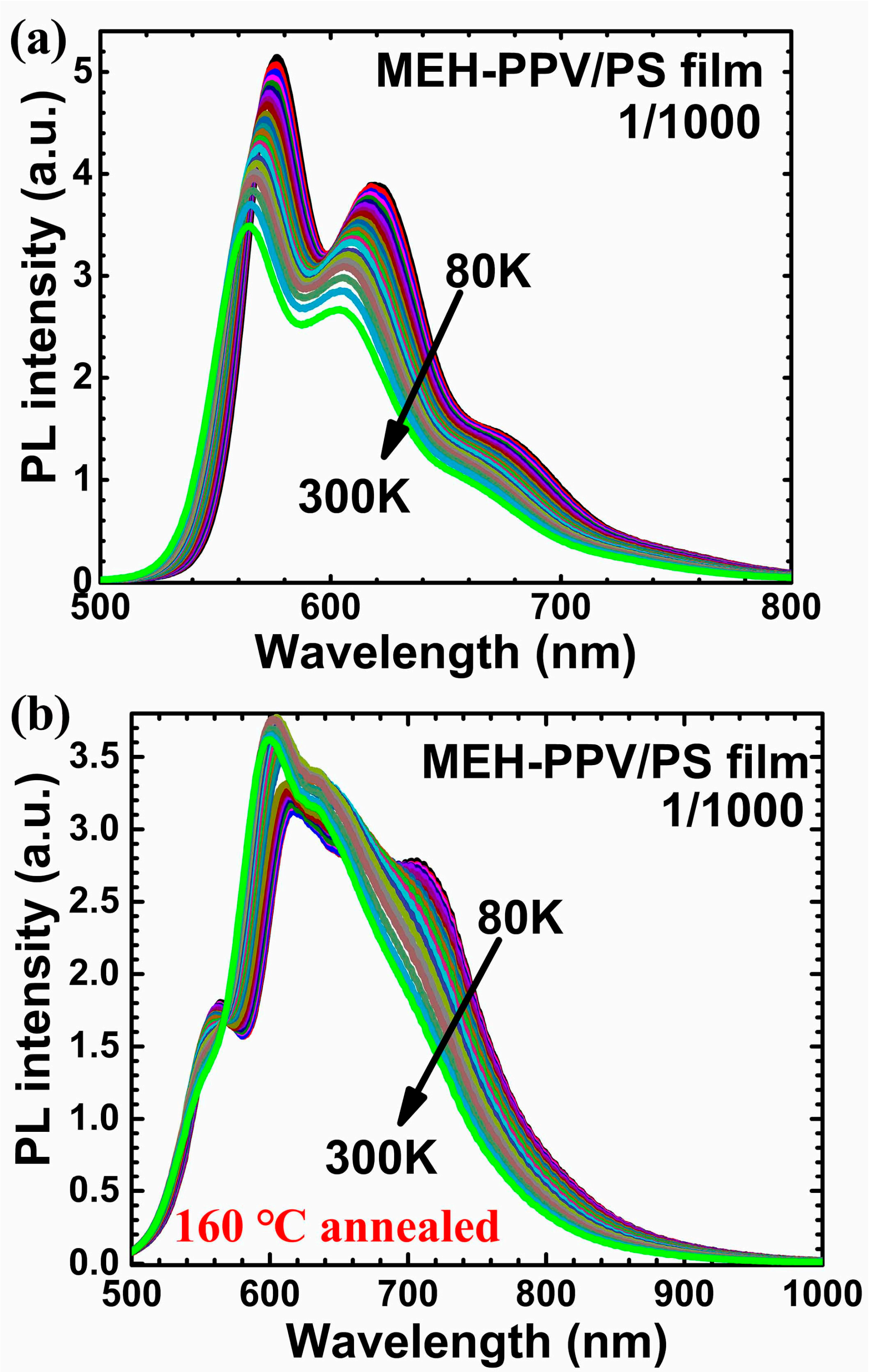

Fig. S11 The temperature dependent photoluminescence spectra of MEH-PPV/PS blends film with the weight ratio of 1/1000 (**a**) as-cast, (**b**) annealed at 160 °C.

References

1. Ostroverkhova, O., Organic Optoelectronic Materials: Mechanisms and Applications. *Chem. Rev.* **2016,** 116, 13279-13412.
